# Change in cognitive performance during seven-year follow-up in midlife is associated with sex, age, and education – The Cardiovascular Risk in Young Finns Study

**DOI:** 10.1007/s00415-024-12466-2

**Published:** 2024-06-02

**Authors:** Marja A. Heiskanen, Jaakko Nevalainen, Katja Pahkala, Markus Juonala, Nina Hutri, Mika Kähönen, Eero Jokinen, Tomi P. Laitinen, Päivi Tossavainen, Leena Taittonen, Jorma S. A. Viikari, Olli T. Raitakari, Suvi P. Rovio

**Affiliations:** 1 Research Centre of Applied and Preventive Cardiovascular Medicine, University of Turku, Kiinamyllynkatu 10, FI-20520 Turku, Finland; 2 Centre for Population Health Research, University of Turku and Turku University Hospital, Turku, Finland; 3 Unit of Health Sciences, Tampere University, Tampere, Finland; 4 Paavo Nurmi Centre, Unit for Health and Physical Activity, University of Turku, Turku, Finland; 5 Department of Medicine, University of Turku and Division of Medicine, Turku University Hospital, Turku, Finland; 6 Tampere Centre for Skills Training and Simulation, Tampere University, Tampere, Finland; 7 Department of Clinical Physiology, Tampere University Hospital and Faculty of Medicine and Health Technology, Tampere University, Tampere, Finland; 8 Department of Pediatric Cardiology, Hospital for Children and Adolescents, Helsinki University Hospital and University of Helsinki, Helsinki, Finland; 9 Department of Clinical Physiology, University of Eastern Finland and Kuopio University Hospital, Kuopio, Finland; 10 Department of Pediatrics, Research Unit of Clinical Medicine, MRC Oulu, University of Oulu, Oulu, Finland; 11 Department of Children and Adolescents, Oulu University Hospital, Oulu, Finland; 12 Department of Pediatrics, Tampere University Hospital, Tampere, Finland; 13 Department of Clinical Physiology and Nuclear Medicine, Turku University Hospital, Turku, Finland; 14 Department of Public Health, University of Turku and Turku University Hospital, Turku, Finland

**Keywords:** Cognitive aging, Midlife, Sex, Education, CANTAB, Longitudinal study

## Abstract

**Objective:**

Sex, age, and education are associated with the level of cognitive performance. We investigated whether these factors modulate the change in cognitive performance in midlife by leveraging the longitudinal data from the Cardiovascular Risk in Young Finns Study (YFS).

**Methods:**

Participants of the YFS cohort performed a computer-based Cambridge Neuropsychological Test Automated Battery (CANTAB) in 2011 and 2018 (n = 1671, age 41–56 years in 2018). Overall cognitive performance and domains representing learning and memory, working memory, reaction time, and information processing were extracted by common principal component analysis from the longitudinal cognitive data. Linear models adjusted for baseline cognitive performance were used to study the association of sex, age, and education with changes in overall cognitive performance and in the cognitive domains.

**Results:**

Cognitive performance decreased in all domains (overall cognition -0.56 SD, *p* < 0.001; working memory -0.81 SD, *p* < 0.001; learning and memory -0.70 SD, *p* < 0.001; reaction time -0.06 SD, *p* = 0.019; information processing -0.03 SD, *p* = 0.016). The decrease in working memory and information processing was greater in females compared to males. Cognitive performance decreased more in older participants in all domains. Education alleviated the decrease in cognitive performance in all domains except reaction time. The beneficial effect of education was greater for males.

**Conclusions:**

This study describes the natural course of aging-related changes in cognitive performance in midlife, the critical time window for early prevention of clinical cognitive decline. These findings provide a reference for studies focusing on determinants of pathological cognitive decline deviating from normal changes in cognitive performance.

**Supplementary Information:**

The online version contains supplementary material available at 10.1007/s00415-024-12466-2.

## Introduction

Preservation of cognitive performance at older age is a fundamental element of healthy aging and quality of life [1]. The disease processes related to cognitive impairment may begin even decades before the cognitive deficits become clinically detectable and progress slowly along a continuum [2–5]. Therefore, studying the determinants contributing to the change in cognitive performance before or at the early stage of the continuum may offer means to preserve optimal cognitive performance as long as possible.

Educational attainment is one of the most commonly used proxies of cognitive reserve [6, 7]. The cognitive reserve hypothesis postulates that individual differences in the cognitive processes or neural networks allow some people to cope better than others with aging-related neuronal loss [8]. The active model of cognitive reserve suggests that e.g. education, occupation, and participation in cognitively stimulating leisure activities could delay the onset of dementia by providing a buffer against the clinical effects of brain damage [8, 9]. While the evidence on the protective effect of education on the level of cognitive performance is convincing [9, 10], the literature regarding the effect of education on the change in cognitive performance is mixed and mostly based on populations of older adults [10–16]. A recent meta-analysis found a wide heterogeneity in the effects of education on the change in cognitive performance, which itself may be an important finding [11]. While the heterogeneity of the results may be related to study design, such as the differing age of the participants, duration of the follow-up period, and cognitive test used, heterogeneity may also reflect differences in societies and whether education is equally accessible for everyone or based on privilege.

Cognitive performance is also modified by sex, which may originate both from biological differences between the sexes as well as from sociocultural norms related to different genders [17–19]. The male and female brains are structured differently [20]. Males typically perform better on visuospatial tasks, whereas females excel at verbal memory tasks [17, 18, 20–22]. One biological explanation for the sex difference is attributed to sex hormones, which regulate brain development and function [17]. Alzheimer’s disease and dementia are more frequent in females compared to males [17, 19, 23, 24], which may partly be explained by biological factors, such as menopause which causes a relatively rapid loss of ovarian sex hormones in females, whereas men’s testosterone levels decline more gradually [18]. Longitudinal studies in older populations have shown inconsistent sex differences in the change of cognitive performance, either reporting a steeper annual decrease in men [25], women [22], or no sex difference [26]. Sociocultural factors, such as accessibility of education, may also drive the sex- or gender-related differences in cognitive decline [23, 24, 27].

The Cardiovascular Risk in Young Finns Study (YFS) is an ongoing epidemiologic study that has followed a population-based cohort of individuals from childhood to adulthood since 1980 [28]. As part of the follow-up studies in 2011 and 2018, cognitive performance was assessed with the Cambridge Neuropsychological Test Automated Battery (CANTAB) including tests that reflect four cognitive domains: learning and memory, working memory, reaction time, and information processing. We have shown that among 34- to 49-year-old participants in 2011, the level of cognitive performance was lower among participants with older age, while education was associated with a higher level of cognitive performance in all of the measured cognitive domains. Males had higher levels of cognitive performance in all cognitive domains except learning and memory, in which females outperformed males [29].

The purpose of the present study is to investigate the change in cognitive performance during a seven-year follow-up period among middle-aged Finns and to study whether age, sex, and education modulate the observed change in cognitive domains. While cognitive deficits are rare at this age range, studying the aging-related cognitive changes already in midlife provides insight into the natural course of change in cognitive performance during the critical time window for shaping the cognitive trajectory towards older age. Ultimately, this study may contribute to the development of tools for early prevention of clinical cognitive decline.

## Methods

### Participants

This study is part of the YFS, which is an ongoing longitudinal population-based study originally focusing on cardiovascular risk factors from childhood to adulthood. The study was designed as a national collaborative effort between all university hospitals and several other institutions in Finland. The first cross-sectional study of the YFS was performed in 1980, and it included 3596 randomly selected children and adolescents (both boys and girls) from six age cohorts (3, 6, 9, 12, 15, and 18 years). Until 2018, the cohort had been regularly followed up in 3- to 9-year intervals. More detailed information on the YFS population and protocol is reported elsewhere [28].

### Cognitive performance

The CANTAB was used to assess cognitive performance among the participants in the two latest follow-up studies conducted in 2011 and during 2018–2020 (hereafter referred as the year 2018 data). The CANTAB is a computerized, predominantly nonlinguistic, and culturally neutral test focusing on a wide range of cognitive domains. The test is performed using a validated touchscreen computer system. The full test battery includes 25 individual tests from which a suitable test battery for each particular study may be selected. In the YFS, the test battery was selected so that it could be accomplished in 20–30 min and included tests that are sensitive to aging [30, 31]. The tests measured several cognitive domains: (a) short-term memory, (b) spatial working memory, (c) problem-solving, (d) reaction time, (e) attention, (f) rapid visual processing, (g) visual memory, (h) episodic memory, and (i) visuospatial learning. Cognitive testing was performed during the clinical examination. Due to the blood sampling included in the study protocol, the participants came to the examinations after fasting for at least 4 h. They were instructed to avoid smoking and heavy physical activity as well as drinking alcohol and coffee during the previous evening and the morning before the examinations. Before the cognitive testing, the participants were provided with a light snack, including a whole grain oat-based snack biscuit, a small portion of fruit or berry oatmeal or oatdrink, and weak fruit or berry juice.

During cognitive testing, the participants first conducted *the Motor Screening Test (MOT)* measuring psychomotor speed and accuracy. In this study, the MOT was considered a training procedure where the participants were introduced to the equipment used in the testing and a screening tool to point out any difficulties in vision, movement, comprehension, or ability to follow simple instructions. During the MOT, a series of red crosses were shown in different locations on the screen, and the participants were advised to touch, as quickly as possible, the center of the cross every time it appeared. *The Paired Associates Learning (PAL) test* was used to assess visual and episodic memory as well as visuospatial associative learning, containing aspects of both a delayed-response procedure and conditional learning (hereafter learning and memory). During the PAL test, 1, 2, 3, 6, or 8 patterns (2, 4, 6, 8, or 12 patterns in year 2018 test) were displayed sequentially in boxes placed on the screen. After that, the patterns were presented in the center of the screen, and the participants were supposed to point to the box in which the particular pattern was previously seen. The test moves on to the next stage if all the patterns are placed in the right boxes. In the case of an incorrect response, all the patterns are redisplayed in their original locations and another recall phase is followed. The test terminated if the patterns were still incorrectly placed after 10 presentation and recall phases (4 in year 2018 test). *The Spatial Working Memory (SWM) test* was used to measure the ability to retain spatial information and to manipulate items stored in the working memory, problem-solving, and the ability to conduct a self-organized search strategy (hereafter working memory). During this test, the participants were presented with either 4, 5, 6, 7, or 8 (3, 4, 6, 8, or 12 boxes in year 2018 test) randomly distributed colored boxes on the screen. After that, the participants were supposed to search for tokens hidden in the boxes. When a token was found, it was supposed to be moved to fill an empty panel on the right-hand side of the screen. Once the token had been moved from the box, the participant had to recall that the computer would never hide a new token in a box that previously contained one; therefore, the participants were not supposed to revisit the same boxes again. *The reaction time (RTI) test* assessed the speed of response and movement on a task where the stimulus was unpredictable (five-choice location task) (hereafter reaction time). In the RTI, five large circles were presented on the screen. The participant was supposed to press down a touchscreen button at the bottom of the screen and wait until a small yellow spot appeared in any of the five large circles. When the yellow spot appeared, the participant was supposed to touch the yellow spot as soon as possible with the same hand that was pressing the touchscreen button. *The Rapid Visual Information Processing (RVP) test* was used to assess, visual processing, recognition, and sustained attention (hereafter information processing). In this test, the participants were presented with three number sequences (3–5–7, 2–4–6, and 4–6–8) next to a large box where numbers 1–9 appeared in a random order at a rate of 100 numbers per minute. Whenever any of the particular sequences were presented, the participant was supposed to press a touchscreen button. Altogether nine target sequences were presented in every 100-s interval during the six-minute assessment phase. During the practice phase, the participant was given visual cues (*i.e.,* colored or underlined numbers) to help recognize the particular sequence. At the assessment phase, the cues were no longer presented.

The implementations of learning and memory and working memory tests were slightly changed between years 2011 and 2018. In the learning and memory test, the number of displayed patterns was increased from 1–8 patterns to 2–12 patterns in 2018 test compared to 2011 test, while the number of recall phases was reduced from 10 to 4 attempts. In the working memory test, the number of colored boxes changed from 4–8 boxes to 3–12 boxes. Thus, 8 variables in the learning and memory test and 7 variables in the working memory test were not comparable between the study years. To overcome slight differences in the implementations of the tests, we used two-phase calibration models on the current and additional data. The models performed well as assessed by comparison to external reference distribution and close internal review of parameters and output. This was done for each of the 15 outcome measures for learning and memory and working memory tests before entering the raw data into the common principal component analysis.

### Common principal component analysis

We have shown previously using the YFS cognitive performance data collected in 2011 that principal component analyses can summarize the rich raw data collected with the CANTAB by reducing redundant information and producing a single test score for each of the cognitive domains as well as for the overall cognition [29]. We used Flury’s common principal component analysis [32] to derive the principal component scores for (i) across all domains / whole CANTAB test battery and (ii) separately for each measured cognitive domain / each of the four separate subtests. As the motor screening test reached a ceiling effect among our study population, all the outcome measures of this test were removed before the common principal component analysis and excluded from all analyses. The main idea of the method is to conduct a principal component analysis for a data set arranged in multiple groups. It allows the groups to have different means, variances, and correlations but assumes that the principal components (eigenvectors) are the same in those groups. In the present study, groups were defined as the year of the CANTAB test performed (2011 and 2018). We standardized the variables of cognitive performance to mean value of 0 and standard deviation of 1 before the analysis and subsequently, windsorized a few outlying values (> 10 SDs from the mean) to ± 10 to control any disproportionate influence. We took the first principal components to subsequent analysis because they represented the majority of the variability in all the domains. For both time points, each of the five principal components (overall cognition, memory and learning, reaction time, information processing, and working memory) were standardized to mean value of 0 and standard deviation of 1 based on the year 2011 data. The change in cognitive performance was calculated by subtracting participants’ standardized year 2018 principal component scores from the standardized year 2011 principal component scores (Δ change). The analysis was implemented with the multigroup package in R (version 4.1.3).

### Age and education

Age was defined in full years at the end of 2018. Education was assessed with questionnaires during all follow-up studies. Total years of education was determined as a continuous variable from self-reported data concerning total years of education until 2018. For stratified analyses, the participants were divided into two education categories using the median of the years of education (15 years) as a cutoff point. Participants with less than 15 years of education formed the low education group, whereas participants with at least 15 years of education were assigned to the high education group. In addition to education, we considered gross yearly income as an indicator of socioeconomic status. Gross income was determined using a self-reported questionnaire in 2018 in which participants were asked to state their gross yearly income in 5000 € intervals (the highest category > 100 000 €/year).

### Menopause and illnesses

Menopausal status was determined using a self-reported questionnaire in 2018 in which females were asked to select the current menopausal status from three categories: pre-menopause (no symptoms), peri-menopause (symptoms including irregular menstruation or hot flushes or night sweats), and post-menopause (menstruation ceased at least one year ago). Prevalence of illnesses was assessed using self-reported questionnaires, in which participants responded whether a medical doctor had diagnosed the given condition by the follow-up at the year 2018. The illnesses reported were: cardiovascular disease (cardiac infarction, coronary heart disease, hypertension, insufficiency of heart, atrial fibrillation, other arrhythmia, valvular defect, congenital heart defect, dilation of aorta, constriction of carotid artery, and/or claudication), brain disease (cerebral thrombosis, cerebral hemorrhage, and/or cerebrovascular accident in the past.), type 2 diabetes, cancer, migraine, depression, and anxiety or other mental disorder. Participants whose body mass index was greater than 30 m^2^/kg in 2018 were considered obese.

### Statistical analyses

The Δ changes of participants’ principal component scores were normally distributed by visual inspection for all cognitive domains. The mean and standard deviation are calculated for the Δ changes in cognitive domains between the year 2011 and the year 2018 (in Online Resource 1 for year 2011 and 2018 standardized principal component scores, respectively). A one-sample Student’s t test was used to study whether the change in cognitive performance is different from zero. A two-sample Student’s t test was used to study the difference of change in cognitive performance between females and males and in stratified analyses. Associations between two categorical variables were studied with the chi-square test. The one-way analysis of variance (ANOVA) with Tukey’s post-hoc tests when appropriate was used to evaluate differences in change in cognitive performance in age cohorts. Pearson’s correlation was used to study the correlations between the change in cognitive performance and education and between the participants’ principal component scores of cognitive domains in the year 2011 and the year 2018.

Multiple linear models were constructed to investigate the associations between the change in principal component scores of cognitive performance with sex, age, and education by using models including sex, age, education, and the year 2011 principal component scores for the respective cognitive domain in the same model. The association of illnesses with the change in cognitive performance was studied by adding them into these models. When evaluating the association of menopause with cognitive performance, a categorical variable describing menopausal status was included in the models. When investigating the role of gross yearly income, it was added into the models as a continuous variable. Finally, interactions between sex, age, and education were investigated by introducing second-order interaction terms separately into the models containing all the main effects. Model residuals were homoscedastic and normally distributed by visual inspection.

The statistical analyses were performed using R (v. 4.2.1, R Foundation for Statistical Computing, Vienna, Austria; https://www.R-project.org/), and the level of statistical significance was set at 0.05.

## Results

### Characteristics of the study population and change in cognitive performance

**Fig. 1:**
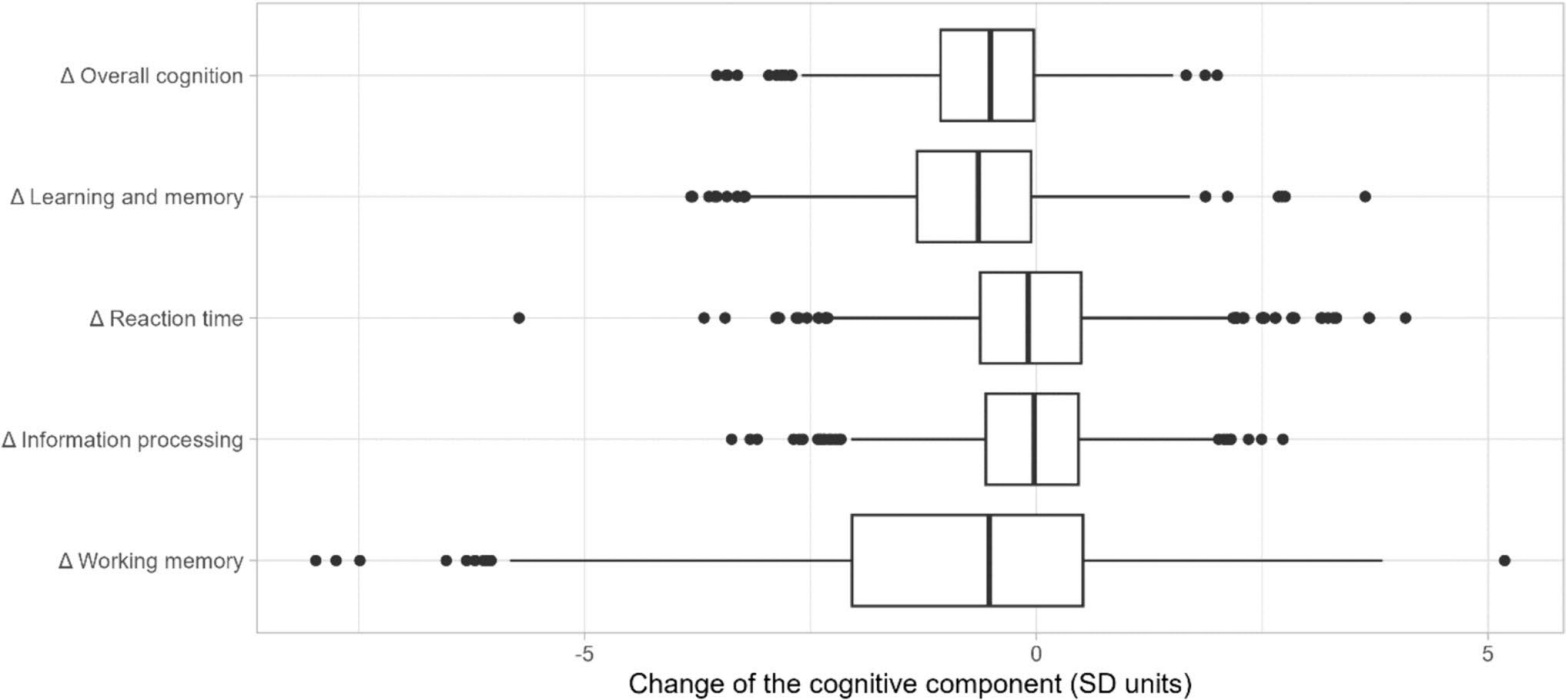
Visualization of the change in the cognitive performance between years 2011 and 2018

During the latest two YFS follow-ups, n = 2025 participants performed cognitive testing in the year 2011 and *n* = 2030 participants in the year 2018. Cognitive data has missing values due to (i) technical reasons (*n* = 176 in 2011; *n* = 13 in 2018), (ii) participant’s unwillingness to participate in some of the tests (*n* = 14 in 2011; *n* = 124 in 2018), (iii) distraction caused by a study nurse or the environment (*n* = 0 in 2011, *n* = 5 in 2018), (iv) unknown reason (*n* = 2 in 2011, *n* = 20 in 2018). From these participants, *n* = 1671 participated in the cognitive testing at both time points and provided longitudinal cognitive data reported in this study. The test-specific numbers of participants and background characteristics are presented in Table 1. The change in cognitive performance in different domains is visualized in Fig. 1 and the mean values of cognitive domains in 2011 and 2018 are presented in Online Resource 1. Overall cognitive performance decreased by -0.56 SD units (*p* < 0.001; on average -0.080 SD units/year) during seven years of follow-up. Working memory decreased by -0.81 SD units (*p* < 001; -0.115 SD units/year), learning and memory by -0.70 SD units (*p* < 0.001; -0.100 SD units/year), reaction time by -0.06 SD units (*p* = 0.019; -0.009 SD units/year), and information processing by -0.03 SD units (*p* = 0.016; -0.005 SD units/year). Year 2011 and 2018 results correlated significantly in each of the cognitive domains (*r* ≥ 0.47, *p* < 0.001 for all domains; data not shown). The coefficients on the first principal components are reported in Online Resource 2 for overall cognition as well as for different cognitive domains. Overall cognition was mostly driven by the test variables related to learning and memory and information processing.

**Table 1:** Background characteristics (year 2018) and the change in cognitive performance from 2011 to 2018 for males and females

	Females	Males	*p* values		
	(*n* = 931)	(*n* = 740)	Females, change	Males, change	Females vs. males
Background characteristics					
Age, years	49.0 (4.9)	48.8 (5.1)	-	-	0.47
Education, years	16.3 (3.7)	15.1 (3.5)	-	-	** < 0.001**
Change in cognitive performance					
Δ Overall cognition (*n* = 1416)	-0.57 (0.77)	-0.55 (0.81)	** < 0.001**	** < 0.001**	0.73
Δ Learning and memory (*n* = 1488)	-0.66 (0.95)	-0.73 (0.99)	** < 0.001**	** < 0.001**	0.13
Δ Reaction time (*n* = 1498)	0.03 (0.90)	-0.16 (0.96)	0.42	** < 0.001**	** < 0.001**
Δ Information processing(*n* = 1578)	-0.07 (0.82)	-0.03 (0.81)	**0.017**	0.34	0.36
Δ Working memory (*n* = 1661)	-0.96 (1.80)	-0.60 (1.87)	** < 0.001**	** < 0.001**	** < 0.001**

Values of cognitive performance are mean changes in SD units and their standard deviations. Δ: Delta change of cognitive performance between years 2018 and 2011. A one-sample Student’s t test was used to study whether the change is different from zero for females and males, respectively. A two-sample Student’s *t* test was used to study the difference between females and males. Statistically significant results are bolded.

### Association with sex

Overall cognition decreased similarly for both sexes during the seven-year follow-up period (Table 1). For females, all cognitive domains except reaction time decreased, while for males, all cognitive domains decreased except working memory, which remained unchanged. When comparing the changes between females and males, reaction time and working memory changed differently; reaction time decreased only in males and working memory only in females.

### Association with age

**Fig. 2:**
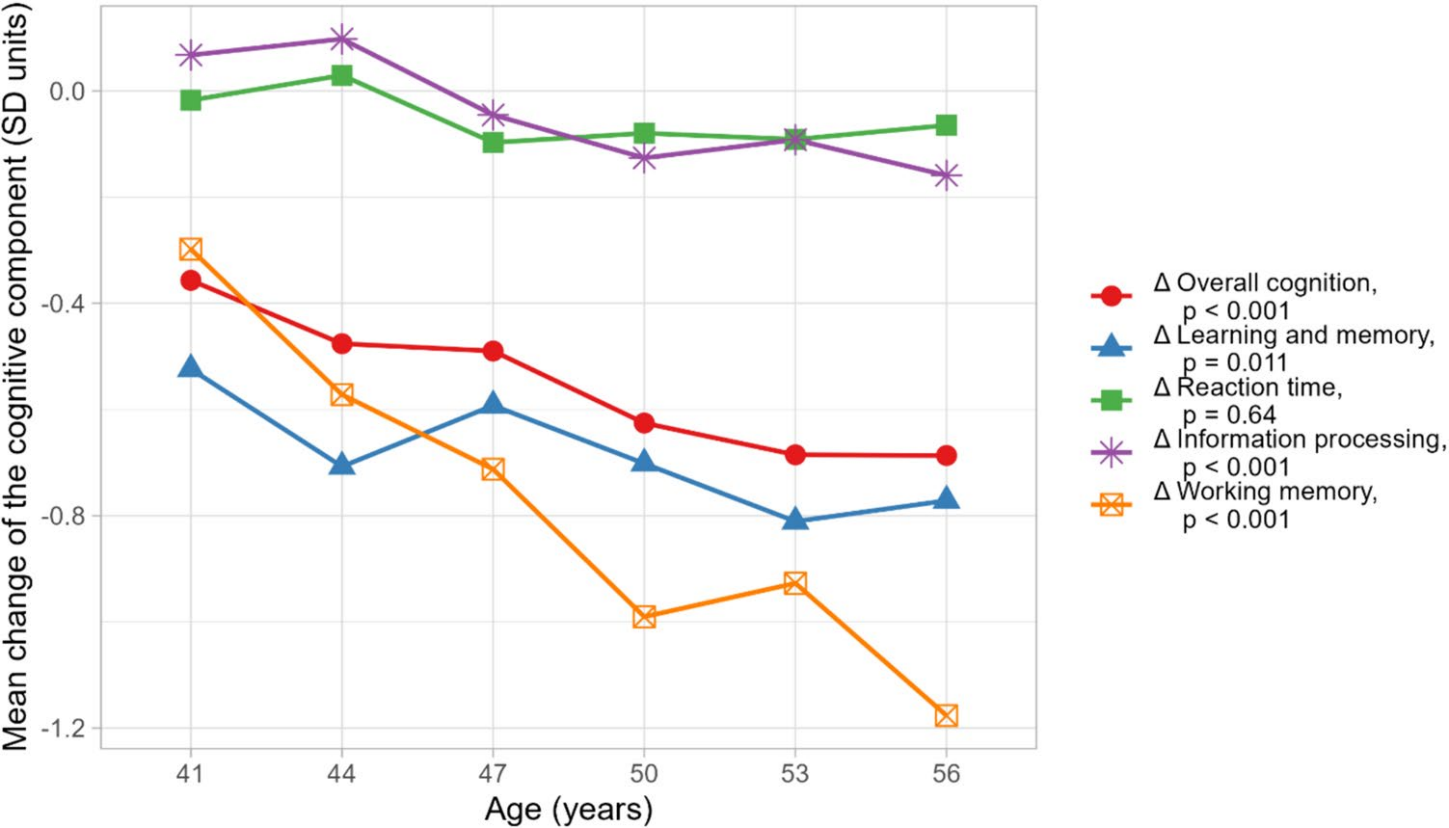
Association between the change in the cognitive performance and age at 2018. A one-way analysis of variance (ANOVA) test was used to evaluate whether there is a difference in the mean changes of cognitive performance between the age groups within overall cognition and within each domain (*p* values are shown in the legend)

The mean changes in the cognitive domains were different between the age cohorts for all the other cognitive domains except reaction time, which remained the same (Fig. 2). The decrease in all cognitive domains except reaction time was more pronounced for ages 50–56 years (in 2018) compared to the reference group of 41-year-old individuals (Online Resource 3).

### Association with education

Years of education was positively correlated with the change in overall cognition (*r* = 0.13, *p* < 0.001), implying that longer education is associated with a smaller decrease in overall cognition. Similarly, positive correlations were found for learning and memory (*r* = 0.078, *p* = 0.003), information processing (*r* = 0.13, *p* < 0.001), and working memory (*r* = 0.062, *p* = 0.014). However, the change in reaction time was not correlated with years of education (*r* = -0.007, *p* = 0.81).

### Multiple linear models

In the multivariate analysis, male sex had a positive association with the change in information processing (+ 0.13 SD units higher for males, *p* = 0.002; Table 2), as well as on working memory (+ 0.43 SD units higher for males, *p* < 0.001).

**Table 2:** Associations between change in cognitive performance, sex, age, and education adjusted for year 2011 result for the respective cognitive domain

	β estimate	95% confidence interval	p value	R^2^
Δ Overall cognition				0.076
(Intercept)	0.24	-0.23, 0.71	0.318	
Sex, male	0.06	-0.02, 0.14	0.145	
Age, years	-0.03	-0.04, -0.02	** < 0.001**	
Education, years	0.04	0.02, 0.05	** < 0.001**	
Overall cognition 2011	-0.17	-0.21, -0.13	** < 0.001**	
Δ Learning and memory				0.094
(Intercept)	0.32	-0.24, 0.88	0.259	
Sex, male	-0.09	-0.18, 0.01	0.086	
Age, years	-0.03	-0.04, -0.02	** < 0.001**	
Education, years	0.03	0.01, 0.04	** < 0.001**	
Learning and memory 2011	-0.29	-0.34, -0.24	** < 0.001**	
Δ Reaction time				0.39
(Intercept)	1.05	0.62, 1.49	** < 0.001**	
Sex, male	-0.04	-0.12, 0.04	0.342	
Age, years	-0.02	-0.03, -0.02	** < 0.001**	
Education, years	0.01	-0.00, 0.02	0.251	
Reaction time 2011	-0.59	-0.63, -0.55	** < 0.001**	
Δ Information processing				0.10
(Intercept)	0.14	-0.32, 0.59	0.554	
Sex, male	0.13	0.05, 0.21	**0.002**	
Age, years	-0.02	-0.03, -0.01	** < 0.001**	
Education, years	0.04	0.03, 0.05	** < 0.001**	
Information processing 2011	-0.23	-0.27, -0.19	** < 0.001**	
Δ Working memory				0.038
(Intercept)	1.22	0.16, 2.27	**0.024**	
Sex, male	0.43	0.24, 0.62	** < 0.001**	
Age, years	-0.06	-0.08, -0.04	** < 0.001**	
Education, years	0.03	0.01, 0.06	**0.009**	
Working memory 2011	-0.13	-0.22, -0.03	**0.010**	

Δ: Delta change of cognitive performance between years 2018 and 2011. Values are β estimates and their 95% confidence intervals and *p* values from linear models. All variables are entered simultaneously into the model. *R*^*2*^ values represent the goodness of fit of each full model. Statistically significant *p* values are bolded.

Age was associated with a decrease in overall cognition (-0.03 SD units/year, *p* < 0.001; Table 2). A similar association was found for all cognitive domains. On the other hand, years of education had the opposite association of the same magnitude as age on overall cognition (+ 0.04 SD units/year of education, *p* < 0.001; Table 2). Similarly, education was directly associated with change in learning and memory, working memory, and information processing. Reaction time was not associated with education.

As the changes in working memory and information processing were associated with sex, we further investigated whether menopause is associated with these changes. In information processing, the decrease was more pronounced in post-menopause (-0.19 SD units smaller in post-menopause compared to pre-menopause, *p* = 0.022; Online Resource 4). However, menopause was not associated with the decrease in working memory.

Some of the participants had developed illnesses by the year 2018 follow-up, which are reported in Online Resource 5. However, after adding the illnesses into the linear models, estimates of age, sex, and education remained essentially the same, and illnesses had only a subtle effect on the change in cognitive performance in midlife (Online Resource 6).

Finally, to study the role of education in the context of participants’ profession and socioeconomic status, we included gross yearly income into the linear models. While gross income was positively associated with the change in reaction time and information processing, the estimates of age, sex, and education remained essentially the same as in the models without adjusting for gross income for all cognitive domains (Online Resource 7).

**Fig. 3:**
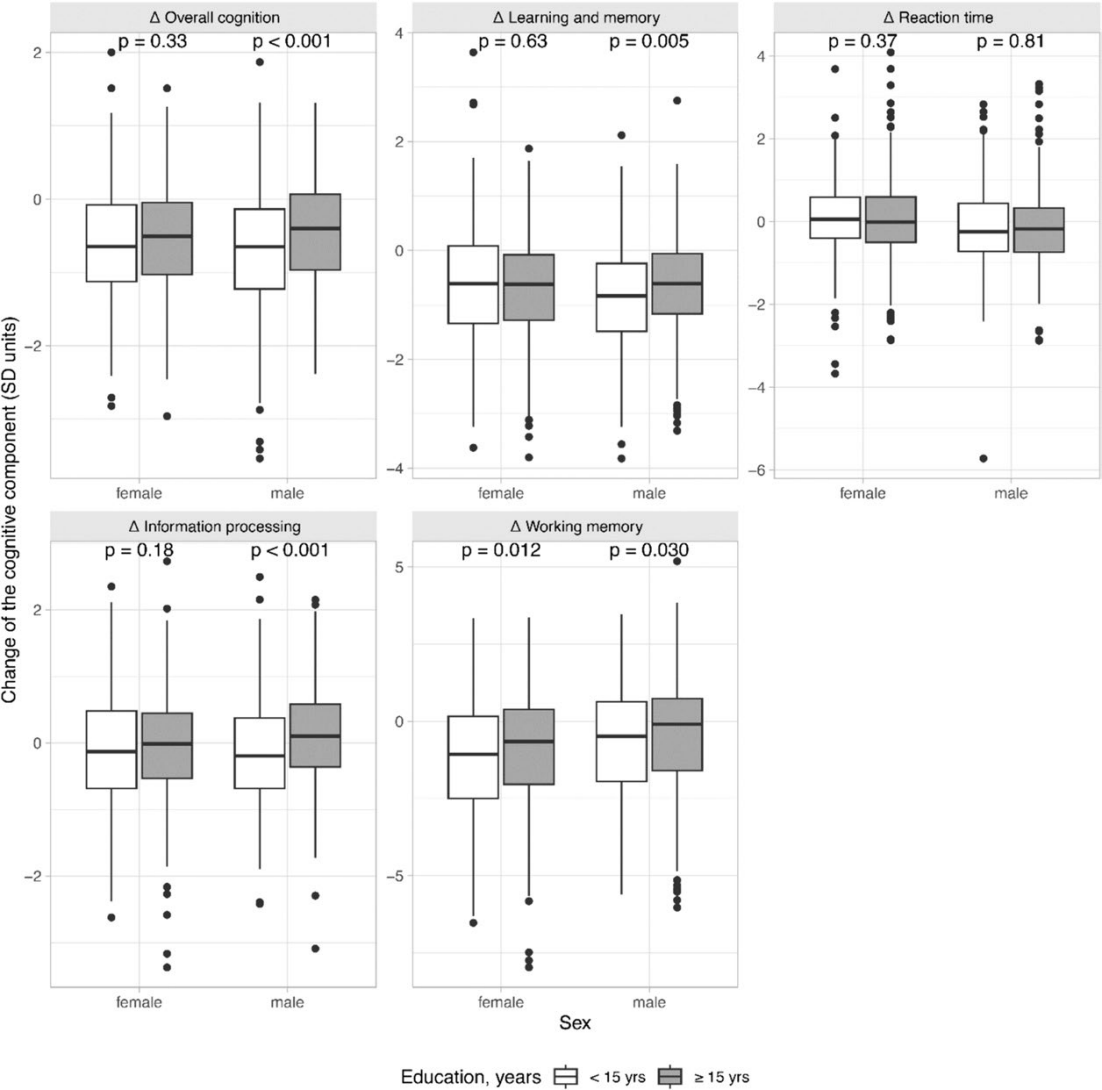
Interaction effect of sex and education on the change in the cognitive components. The interaction effect is statistically significant for the change in overall cognition (*p* = 0.003), learning and memory (*p* = 0.006), and information processing (*p* = 0.014), implying that the effect of education is different for males and males in these domains. The difference between high (≥ 15 years) and low (< 15 years) education was investigated using Student’s *t* test for each sex, and corresponding *p* values are shown in the figure

Interactions of sex, age, and education were investigated by adding interaction terms for each combination of two variables (sex × age, sex × education, age × education) separately into the multiple linear models described in Table 2. Interactions between sex and age or between age and education were not statistically significant for any of the cognitive domains, nor for overall cognitive performance (data not shown). However, a significant interaction between sex and education was found for overall cognition (*p* = 0.003), learning and memory (*p* = 0.006), and information processing (*p* = 0.014). The interaction effect of sex and education on the change of the cognitive domains was further investigated by stratifying with educational level and visualized in Fig. 3. For overall cognition, learning and memory, and information processing, the decrease of these cognitive domains was alleviated in males with a higher educational level, whereas for females, the level of education did not modify the decrease in the respective cognitive domains. However, in working memory, a higher level of education alleviated the decrease for both sexes (Fig. 3).

## Discussion

Aging populations worldwide pose a growing public health and economic burden due to growing numbers of cognitive deficits. While curative treatments for e.g., Alzheimer’s disease are still waiting to be applied in wide clinical use, early prevention must be targeted to people young enough so that risk factors related to cognitive decline can still be modified. While many studies have investigated the role of education and sex on cognitive performance in older populations, the results may not be totally transferrable to people currently in midlife living in different sociocultural environments compared to previous generations. For instance, the rapid increase of artificial intelligence applications may have an impact on people’s behavior and how they acquire cognitive reserve in the near future, which may be comparable to the Internet becoming part of our everyday life that was not there when older generations were in midlife. Therefore, the aim of the present study is to describe the aging-related cognitive changes in a population currently in midlife, the critical time window for shaping the cognitive trajectory towards older age.

We have previously shown using year 2011 YFS data on cognitive performance, that the level of overall cognition was higher in males compared to females, and the same trend was observed for all the other cognitive domains except learning and memory, in which females outperformed males [29]. In the present study, cognitive performance decreased similarly for both sexes in overall cognition and learning and memory. Information processing and working memory decreased more in females than in males. Previous studies have reported age-related sex and gender differences in cognition [17, 18, 21, 22, 25], but no difference between sexes is also observed [26]. Typical male-like cognition traits include better spatial abilities, whereas female-type cognition includes improved feats of episodic memory and verbal fluency [17, 18, 20–22]. One of the biological factors accounting for differences in cognitive performance throughout the lifespan is sex hormones [17, 19, 23, 24]. A major life event affecting gonadal hormone production occurring in a female’s life is menopause. Compared to a gradual decline of testosterone levels in males, females lose relatively rapidly ovarian sex hormones during menopause [18]. Menopause may also have an impact on organization of functional brain networks [33]. In this study, the decrease in information processing was greater in females whose menstruation had ceased at least one year ago prior to the latest cognitive testing compared to females with a normal menstruation cycle, suggesting that hormonal changes related to menopause may contribute to the change in cognitive performance. Longer life expectancy means that females may live about a third of their lives after menopause, which warrants further studies regarding the effects of sex hormones on cognitive performance. Another major aspect related to sex- or gender-driven differences are sociocultural norms for each gender [22–24]. For instance, in many cultures and societies, education has long been a privilege of males, and females’ possibilities to acquire higher education have been limited. However, in those societies where education has become equally accessible to both sexes, the rate of Alzheimer’s disease in females has declined [18]. In the present study, females had even more years of education compared to males. Thus, the more pronounced decrease in working memory and information processing in females is unlikely related to differences in education.

We have reported before that the level of cognitive performance was lower in the older participants at the baseline when the participants were 34–49 years old [29]. In the present study among 41- to 56-year-old participants, we observed that the decrease in cognitive performance was more pronounced in the older participants, which may reflect the normal aging process in midlife. While some of the study participants have developed illnesses and nine participants had experienced a stroke, the majority of the participants were healthy. When including illnesses in the models, the associations between change in cognitive performance and age, sex, and education remained the same. Nevertheless, it may be that the effects of unhealthy lifestyle choices and underlying diseases not yet clinically detectable start to accumulate in later midlife, accelerating the decline of cognitive performance [34, 35]. However, whether the more pronounced decrease in cognitive performance among older participants in the present study is due to normal aging or affected by other lifestyle factors, our data suggests that a potential target age for early prevention may well be substantially before 60 years of age at which the incidence of cognitive impairment starts to increase [36].

Our data indicates that longer education may alleviate the aging-related decrease in cognitive performance, even after adjusting for gross yearly income. For instance, the beta-estimates of education and age were of similar magnitude but of opposite directions for overall cognition and learning and memory. Speculatively, if education would protect against aging-related cognitive decline, our models suggest that e.g., 10 more years of age would result in a -0.3 SD decrease in memory and learning, which would require 10 more years of education to reverse the effect of aging. Thus, from a practical point of view, the possible protective effect of education against aging-related cognitive decline is limited. The literature supports the positive association between years of education and the level of cognitive performance [9, 10] but the association between education and the change in cognitive performance is controversial [11–16]. A recent meta-analysis concluded that the role of education was negligibly small with regard to change in cognitive performance [11]. However, as the authors pointed out, the heterogeneity of the results was considerable, which may reflect differences in the study design but also differences in societies. The country of residence may also influence the results [10, 12]. While years of education may contribute to the increased cognitive reserve and hence protect against age-related decline of cognitive performance, various tasks related to employment or leisure time activities, such as literacy, may also improve cognitive reserve [8, 9, 37]. For instance, while basic education has been mandatory for everyone in Finland for over a hundred years, the demand for formal educational degrees has been much lower for the older generation now at the retirement to get high-ranking employment compared to modern standards. Therefore, years of formal education may not reflect the acquired cognitive reserve throughout the lifespan in older populations, which may partly explain why the change in cognitive performance has not been related to education in some of the previous studies. Another aspect that may explain the divergence of previous results is the cognitive test used and whether the discriminatory power of the test has been high enough in healthy populations. In the present study, we used a computerized CANTAB test, which is identical for all participants and allows accurate and reliable measurement and recording of, for example, latency times. The CANTAB test has adequate discriminatory power in healthy adults [30, 31], and the only ceiling effect we observed was in the motor screening test measuring psychomotor speed and accuracy, which was considered as an introduction of the testing platform to our healthy participants and excluded from all analyses.

Interestingly, our data suggests that longer education may be even more beneficial for males than for females. For those males who had longer education than the median of 15 years in our study population, the decrease in cognitive performance was alleviated in overall cognition, learning and memory, and information processing. For females, the level of education did not alter the results for these cognitive domains. While we have no data-driven explanation for this phenomenon, we speculate that longer education may increase awareness about lifestyle choices on brain health, especially among males. For instance, European males are generally less aware of the effects of substance use, sleeping habits, and diet on brain health compared to females, whereas respondents with higher education levels and females recognized several lifestyle factors as having a strong influence on brain health [38]. Also, females typically visit a family doctor or primary care more often than males [39, 40]. Hence, it may be that especially among males, higher education increases the awareness of both general and brain health, which may help them to preserve cognitive performance better compared to males with lower education.

The strength of this study is the YFS population, which originally recruited participants from different locations in Finland, representing individuals from both urban and rural areas. Despite the living location, Finnish citizens have equal possibilities to acquire education that is organized by the government and is free of charge even up to a university degree. Hence, the bias related to the availability of education concerning both sex and living location is small compared to many other countries. Another benefit of the present study is the CANTAB test battery, which provides an identical testing procedure for every participant. Hence, e.g., the bias related to an examiner in traditional, noncomputerized tests is reduced. The CANTAB test battery covers a wide spectrum of cognitive domains that are related to brain structures typically altered already in the early stage of cognitive decline [41, 42]. For instance, the memory and learning test assesses cognitive domains related to the medial temporal lobes [43], which is the brain region typically affected first in clinical cognitive impairment [44, 45]. While the CANTAB test is not currently widely used in clinical practice, it could provide a fast and cost-effective method to screen those individuals at risk of cognitive deficits early enough so that risk factors could still be modified.

The study is not without limitations. While the current cognitive test battery allowed to study a wide spectrum of cognitive domains, e.g., the verbal aspects of cognition were not examined. Also, tests related to inhibition and delayed recall were lacking. Verbal fluency and memory are often stronger in females compared to males. It may be that the strongest cognitive domains of females were not examined, which may present some bias to the current results. The test–retest reliability of the CANTAB test battery has been questioned by detecting learning effects within a 3-month retest period in healthy adults [46]. However, many commonly used cognitive neuropsychological tests show similar test–retest reliability [47]. Also, since the follow-up period was seven years in the present study, the learning effect was most likely small, if any. Secondly, we studied only the effect of sex, age, and education on the change in cognitive performance. While these estimates remained essentially the same after adjusting for illnesses, many other factors are also related to cognition, such as diet, physical activity, smoking habits, alcohol consumption, level of engagement in society, social and work-related stressors, caregiving of a parent/spouse with memory disease and marital status, which may affect differently females and males, and hence modulate the risk of cognitive impairment [17, 18, 23, 24]. Illnesses, especially brain diseases such as cerebral thrombosis (stroke), cerebral hemorrhage or past cerebrovascular accident, had only a subtle effect on the results, which is probably due to a small number of diagnosed cases in our middle-aged study population. However, in older populations, the role of illnesses on the change in cognitive performance could be more significant.

To conclude, the present study shows that cognitive performance decreases already in midlife. The decrease in working memory and information processing was larger in females compared to males. Age was associated with a decrease in cognitive performance in all domains. However, education alleviated the decrease in cognitive performance in all other cognitive domains except reaction time. Longer education was even more beneficial for males with regard to overall cognition as well as tasks related to learning and memory and information processing. This longitudinal population-based study describes natural course of the change in cognitive performance in midlife, bringing necessary evidence for studies focusing on determinants of pathological cognitive decline deviating from normal aging-related changes. Optimizing strategies for early prevention could postpone the onset of cognitive impairment in older age and provide as many cognitively healthy years as possible.

### Supplementary Information

Below is the link to the electronic supplementary material.

Supplementary file1 (PDF 635 KB)

## Data Availability

Due to the local legal restrictions concerning the distribution of all personal information, allowance of open access to the YFS data is not possible. Therefore, data sharing outside the study group requires a data-sharing agreement. Investigators can submit an expression of interest to the YFS Steering Group / Data Sharing Committee (PI of the YFS olli.raitakari@utu.fi).
