# Supplementary material for: Change in cognitive performance during seven-year follow-up in midlife is associated with sex, age, and education – The Cardiovascular Risk in Young Finns Study
Source: J Neurol. 2024 Jun 2;271(8):5165–76. doi: 10.1007/s00415-024-12466-2 (PMC11319598; doi:10.1007/s00415-024-12466-2)
Supplement: Supplementary file 1 — Supplementary file1 (PDF 635 KB) [file 415_2024_12466_MOESM1_ESM.pdf]

Supplementary Material

Change in cognitive performance during seven-year follow-up in midlife is associated with sex, age, and education – The Cardiovascular Risk in Young Finns Study

Marja A. Heiskanen*, Jaakko Nevalainen, Katja Pahkala, Markus Juonala, Nina Hutri, Mika Kähönen, Eero Jokinen, Tomi P. Laitinen, Päivi Tossavainen, Leena Taittonen, Jorma S.A. Viikari, Olli T. Raitakari, Suvi P. Rovio

***Address for correspondence:** Marja A. Heiskanen, Research Centre of Applied and Preventive Cardiovascular Medicine, University of Turku, Finland. marja.heiskanen@utu.fi; Tel.: +358-40-738-4663.

Online Resource 1 Cognitive performance in 2011 and in 2018.

	2011	*2018*	*p value 2011 vs. 2018*
All subjects (n = 1671)			
Overall cognition			
Mean (SD)	0.00 (1.00)	-0.56 (1.19)	**<0.001**
Missing	169 (10.1%)	131 (7.8%)	
Learning and memory			
Mean (SD)	0.00 (1.00)	-0.70 (1.22)	**<0.001**
Missing	145 (8.7%)	65 (3.9%)	
Reaction time			
Mean (SD)	0.00 (1.00)	-0.06 (0.85)	**0.019**
Missing	165 (9.9%)	10 (0.6%)	
Information processing			
Mean (SD)	0.00 (1.00)	-0.03 (1.14)	**0.016**
Missing	23 (1.4%)	76 (4.5%)	
Working memory			
Mean (SD)	0.00 (1.00)	-0.81 (2.11)	**<0.001**
Missing	4 (0.2%)	6 (0.4%)	
Males (n = 740)			
Overall cognition			
Mean (SD)	0.02 (1.04)	-0.55 (1.24)	**<0.001**
Missing	69 (9.3%)	38 (5.1%)	
Learning and memory			
Mean (SD)	-0.06 (1.01)	-0.80 (1.23)	**<0.001**
Missing	61 (8.2%)	20 (2.7%)	
Reaction time			
Mean (SD)	0.14 (1.02)	-0.03 (0.88)	**<0.001**
Missing	69 (9.3%)	4 (0.5%)	
Information processing			
Mean (SD)	0.07 (1.01)	0.05 (1.16)	0.34
Missing	7 (0.9%)	21 (2.8%)	
Working memory			
Mean (SD)	0.21 (0.99)	-0.39 (2.10)	**<0.001**
Missing	3 (0.4%)	3 (0.4%)	
Females (n = 931)			
Overall cognition			
Mean (SD)	-0.02 (0.97)	-0.58 (1.15)	**<0.001**
Missing	100 (10.7%)	93 (10.0%)	
Learning and memory			
Mean (SD)	0.05 (0.99)	-0.62 (1.20)	**<0.001**
Missing	84 (9.0%)	45 (4.8%)	
Reaction time			
Mean (SD)	-0.11 (0.97)	-0.09 (0.81)	0.42
Missing	96 (10.3%)	6 (0.6%)	
Information processing			
Mean (SD)	-0.06 (0.99)	-0.10 (1.12)	**0.017**
Missing	16 (1.7%)	55 (5.9%)	
Working memory			
Mean (SD)	-0.16 (0.98)	-1.13 (2.06)	**<0.001**
Missing	1 (0.1%)	3 (0.3%)	

A two-sample paired Student's t test was used to study the difference between years 2011 and 2018. Statistically significant results are bolded.

Online Resource 2 Coefficients of the CANTAB test variables on the first principal components for cognitive performance.

	Overall cognition	Learning and memory	Reaction time	Information processing	Working memory
Variance explained by the first principal component (%)	24.9	59.4	36.7	64.5	33.2
PAL - First attempt memory score	0.339	0.474			
PAL - Mean errors to success	-0.272	-0.343			
PAL - Number of patterns reached	0.067	0.142			
PAL - Total errors (6 patterns, adjusted)	-0.237	-0.358			
PAL - Total errors (8 patterns, adjusted)	-0.262	-0.382			
PAL - Total errors (adjusted)	-0.297	-0.427			
PAL - Total attempts	-0.353	-0.426			
RTI - Error score	0.008		-0.018		
RTI - Mean movement time	-0.090		-0.826		
RTI - Mean reaction time	-0.087		-0.563		
RVP - Mean response latency	-0.168			-0.257	
RVP - A' (sensitivity to target sequence)	0.385			0.602	
RVP - Probability of false alarm	-0.093			-0.105	
RVP - Probability of hit	0.379			0.611	
RVP - Total false alarms	-0.045			-0.052	
RVP - Total hits	0.157			0.250	
RVP - Total misses	-0.214			-0.349	
SWM - Between errors (4 boxes)	-0.058				-0.281
SWM - Between errors (total)	-0.172				-0.655
SWM - Double errors (4 boxes)	-0.007				-0.027
SWM - Double errors (total)	-0.027				-0.193
SWM - Strategy (6-8 boxes)	-0.137				-0.643
SWM - Within errors (4 boxes)	-0.010				-0.021
SWM - Within errors (total)	-0.027				-0.198

PAL: Learning and memory; RTI: Reaction Time; RVP: Information Processing; SWM: Working Memory test. The cell colors visualize the loadings within each cognitive domain so that the values greater than zero are shown in red and smaller than zero in blue.

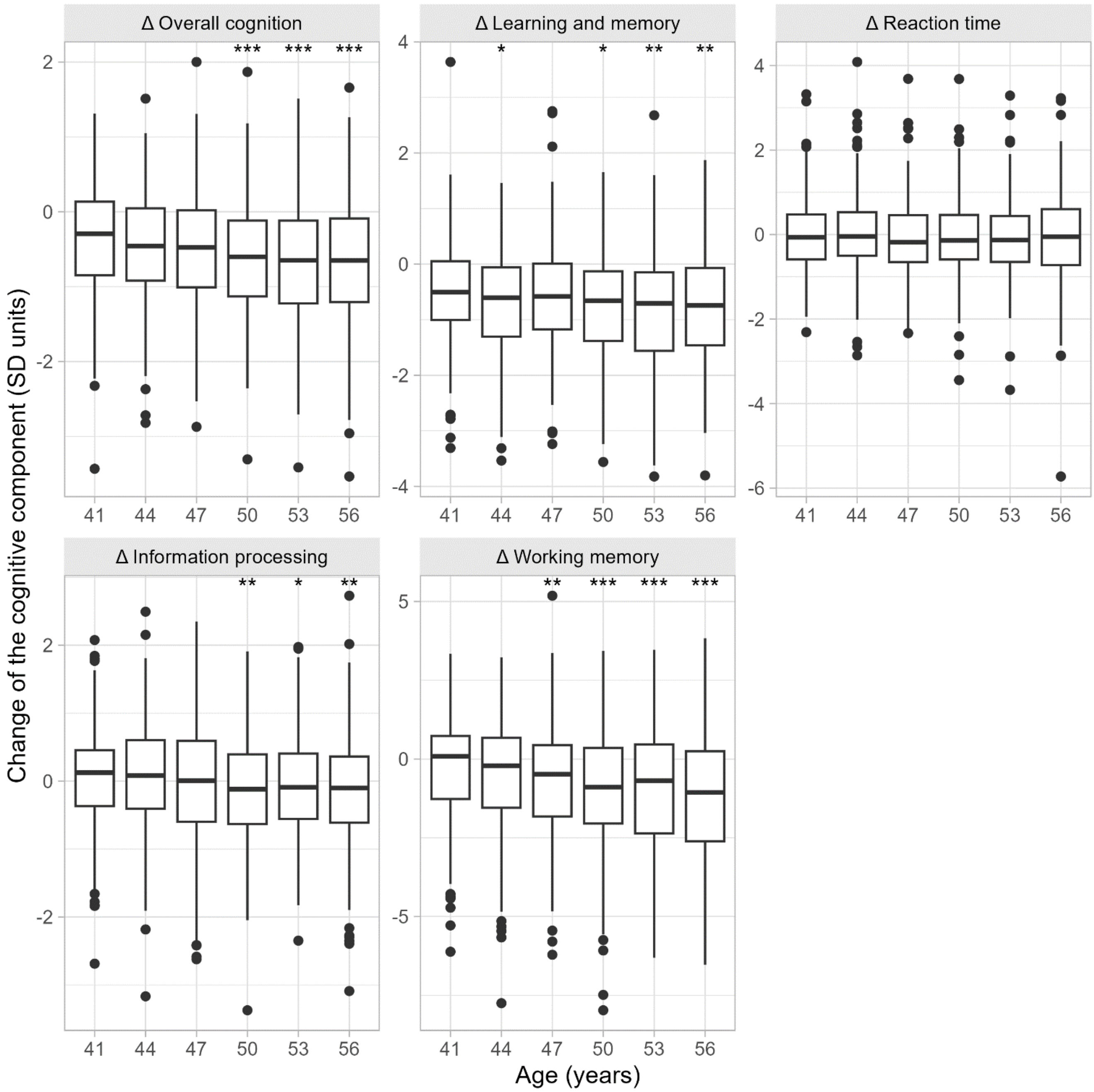

Online Resource 3 Association between the change in the cognitive performance and age. The asterisks indicate the statistical significance of the pairwise comparisons between each age cohort and the reference at age cohort of 41 years. *** $p < 0.001$; ** $p < 0.01$; * $p < 0.05$.

Online Resource 4 Association between change in cognitive performance, age, education, and menopause adjusted for year 2011 result for the respective cognitive domains in female subjects. The effect of menopause on females was tested only for information processing and working memory as these cognitive domains were decreased more in females compared to males (Table 2).

	β estimate	*95% confidence interval*	*p value*	R^2
Δ Information processing				0.10
(Intercept)	-0.11	-0.80, 0.59	0.760	
Age, years	-0.01	-0.02, 0.00	0.213	
Education, years	0.03	0.02, 0.05	**<0.001**	
Menopause, peri	0.03	-0.12, 0.17	0.714	
Menopause, post	-0.19	-0.35, -0.03	**0.022**	
Information processing 2011	-0.23	-0.29, -0.18	**<0.001**	
Δ Working memory				0.025
(Intercept)	0.81	-0.78, 2.40	0.317	
Age, years	-0.05	-0.08, -0.02	**0.002**	
Education, years	0.04	0.00, 0.07	**0.036**	
Menopause, peri	0.13	-0.20, 0.45	0.454	
Menopause, post	0.04	-0.34, 0.41	0.841	
Working memory 2011	-0.11	-0.24, 0.03	0.112	

Δ: Delta change of cognitive performance between years 2018-2020 and 2011.
Menopause was reported in three categories: pre-menopause (no symptoms; reference group), peri-menopause (symptoms including irregular menstruation or hot flushes or night sweats), and post-menopause (menstruation ceased at least one year ago). Statistically significant results are bolded.

Online Resource 5 Prevalence of illnesses in year 2018.

	female (N=931)	*male (N=740)*	*p-value (females vs. males)*
Cardiovascular disease[1,]*			
No	681 (73.1%)	513 (69.3%)	0.45
Yes	225 (24.2%)	186 (25.1%)	
Missing	25 (2.7%)	41 (5.5%)	
Brain disease[2,]*			
No	892 (95.8%)	687 (92.8%)	0.94
Yes	14 (1.5%)	12 (1.6%)	
Missing	25 (2.7%)	41 (5.5%)	
Type 2 diabetes*			
No	866 (93.0%)	660 (89.2%)	0.37
Yes	37 (4.0%)	36 (4.9%)	
Missing	28 (3.0%)	44 (5.9%)	
Cancer*			
No	883 (94.8%)	683 (92.3%)	0.65
Yes	21 (2.3%)	13 (1.8%)	
Missing	27 (2.9%)	44 (5.9%)	
Migraine*			
No	692 (74.3%)	646 (87.3%)	**<0.001**
Yes	212 (22.8%)	49 (6.6%)	
Missing	27 (2.9%)	45 (6.1%)	
Depression*			
No	808 (86.8%)	649 (87.7%)	**0.009**
Yes	96 (10.3%)	47 (6.4%)	
Missing	27 (2.9%)	44 (5.9%)	
Anxiety or other mental disorder[3,]*			
No	844 (90.7%)	655 (88.5%)	0.52
Yes	59 (6.3%)	39 (5.3%)	
Missing	28 (3.0%)	46 (6.2%)	
Obesity[4]			
No	665 (71.4%)	536 (72.4%)	0.69
Yes	266 (28.6%)	204 (27.6%)	

[1]Cardiac infarction, coronary heart disease, hypertension, insufficiency of heart, atrial fibrillation, other arrhythmia, valvular defect, congenital heart defect, dilation of aorta, constriction of carotid artery, and/or claudication.
[2]Cerebral thrombosis, cerebral hemorrhage, and/or cerebrovascular accident in the past.
[3]Anxiety or other mental disorder was asked as a one question in self-reported questionnaire. Therefore, this group includes individuals diagnosed with anxiety as well with other affective and non-affective psychotic disorders and personality disorders.
[4]If body mass index is greater than 30 m^2/kg, participant is considered obese.
*Values are based on self-reported questionnaires of whether a medical doctor has diagnosed the given condition by the year 2018.

Online Resource 6 Association between change in cognitive performance, sex, age, and education adjusted for year 2011 result for the respective cognitive domains and illnesses.

	β estimate	*95% confidence interval*	*p value*	R^2
Δ *Overall cognition*				0.081
(Intercept)	0.22	-0.26, 0.70	0.372	
Sex, male	0.07	-0.02, 0.15	0.126	
Age, years	-0.03	-0.04, -0.02	**<0.001**	
Education, years	0.04	0.02, 0.05	**<0.001**	
Overall cognition 2011	-0.17	-0.21, -0.12	**<0.001**	
Cardiovascular disease[1], yes	-0.02	-0.12, 0.08	0.696	
Brain disease[2], yes	-0.08	-0.43, 0.26	0.630	
Type 2 diabetes, yes	-0.09	-0.31, 0.12	0.405	
Cancer, yes	0.19	-0.10, 0.48	0.201	
Migraine, yes	0.07	-0.05, 0.19	0.228	
Depression, yes	0.02	-0.14, 0.18	0.795	
Anxiety or other mental disorder[3], yes	-0.14	-0.33, 0.05	0.138	
Obesity[4], yes	-0.02	-0.12, 0.08	0.659	
Δ *Learning and memory*				0.095
(Intercept)	0.32	-0.25, 0.90	0.266	
Sex, male	-0.09	-0.19, 0.02	0.101	
Age, years	-0.03	-0.04, -0.02	**<0.001**	
Education, years	0.03	0.01, 0.04	**<0.001**	
Learning and memory 2011	-0.28	-0.33, -0.23	**<0.001**	
Cardiovascular disease[1], yes	0.00	-0.12, 0.12	0.969	
Brain disease[2], yes	-0.29	-0.68, 0.09	0.131	
Type 2 diabetes, yes	0.01	-0.23, 0.26	0.908	
Cancer, yes	0.26	-0.09, 0.61	0.145	
Migraine, yes	0.03	-0.11, 0.17	0.674	
Depression, yes	0.04	-0.14, 0.23	0.641	
Anxiety or other mental disorder[3], yes	-0.08	-0.30, 0.14	0.461	
Obesity[4], yes	-0.01	-0.13, 0.10	0.856	
Δ *Reaction time*				0.39
(Intercept)	0.99	0.54, 1.43	**<0.001**	
Sex, male	-0.01	-0.09, 0.07	0.826	
Age, years	-0.02	-0.03, -0.02	**<0.001**	
Education, years	0.01	-0.00, 0.02	0.230	
Reaction time 2011	-0.59	-0.63, -0.55	**<0.001**	
Cardiovascular disease[1], yes	0.02	-0.08, 0.11	0.750	
Brain disease[2], yes	-0.09	-0.41, 0.22	0.551	
Type 2 diabetes, yes	-0.08	-0.28, 0.12	0.416	
Cancer, yes	0.05	-0.23, 0.32	0.734	
Migraine, yes	0.18	0.07, 0.29	**0.001**	
Depression, yes	-0.03	-0.18, 0.12	0.740	
Anxiety or other mental disorder[3], yes	-0.14	-0.31, 0.03	0.114	
Obesity[4], yes	0.03	-0.06, 0.12	0.551	

Δ *Information processing*				0.11
(Intercept)	0.11	-0.35, 0.58	0.630	
Sex, male	0.15	0.06, 0.23	**0.001**	
Age, years	-0.02	-0.03, -0.01	**<0.001**	
Education, years	0.04	0.03, 0.05	**<0.001**	
Information processing 2011	-0.23	-0.28, -0.19	**<0.001**	
Cardiovascular disease[1], yes	0.01	-0.08, 0.11	0.832	
Brain disease[2], yes	0.11	-0.24, 0.45	0.542	
Type 2 diabetes, yes	-0.15	-0.35, 0.05	0.140	
Cancer, yes	0.09	-0.19, 0.37	0.526	
Migraine, yes	0.13	0.02, 0.24	**0.022**	
Depression, yes	0.00	-0.15, 0.16	0.975	
Anxiety or other mental disorder[3], yes	-0.13	-0.31, 0.05	0.161	
Obesity[4], yes	-0.06	-0.16, 0.03	0.178	
Δ *Working memory*				0.044
(Intercept)	1.05	-0.03, 2.13	0.058	
Sex, male	0.41	0.21, 0.60	**<0.001**	
Age, years	-0.05	-0.07, -0.03	**<0.001**	
Education, years	0.04	0.01, 0.06	**0.003**	
Working memory 2011	-0.13	-0.23, -0.03	**0.009**	
Cardiovascular disease[1], yes	-0.30	-0.52, -0.08	**0.008**	
Brain disease[2], yes	-0.06	-0.80, 0.69	0.880	
Type 2 diabetes, yes	0.19	-0.27, 0.65	0.421	
Cancer, yes	-0.25	-0.89, 0.38	0.436	
Migraine, yes	-0.02	-0.27, 0.24	0.908	
Depression, yes	-0.16	-0.51, 0.20	0.391	
Anxiety or other mental disorder[3], yes	-0.31	-0.73, 0.11	0.146	
Obesity[4], yes	0.04	-0.17, 0.26	0.685	

Δ: Delta change of cognitive performance between years 2018 and 2011. Values are β estimates and their 95% confidence intervals and *p* values from linear models. All variables are entered simultaneously into the model. Variables related to illnesses are defined the same way as in Supplementary Table 4. For illnesses, reference value is "No". R^2 values represent the goodness of fit of each full model. Statistically significant *p* values are bolded.

[1]Cardiac infarction, coronary heart disease, hypertension, insufficiency of heart, atrial fibrillation, other arrhythmia, valvular defect, congenital heart defect, dilation of aorta, constriction of carotid artery, and/or claudication.

[2]Cerebral thrombosis, cerebral hemorrhage, and/or cerebrovascular accident in the past.

[3]Anxiety or other mental disorder was asked as a one question in self-reported questionnaire. Therefore, this group includes individuals diagnosed with anxiety as well with other affective and non-affective psychotic disorders and personality disorders.

[4]If body mass index is greater than 30 m^2/kg, participant is considered obese.

Online Resource 7 Association between change in cognitive performance, sex, age, and education adjusted for gross yearly income in the year 2018 and year 2011 result for the respective cognitive domains.

	β estimate	*95% confidence interval*	*p value*	R^2
Δ *Overall cognition*				0.081
(Intercept)	0.23	-0.24, 0.70	0.339	
Sex, male	0.04	-0.04, 0.13	0.307	
Age, years	-0.03	-0.04, -0.02	**<0.001**	
Education, years	0.03	0.02, 0.04	**<0.001**	
Gross income, 5000 €	0.01	-0.00, 0.02	0.068	
Overall cognition 2011	-0.18	-0.23, -0.14	**<0.001**	
Δ *Learning and memory*				0.095
(Intercept)	0.28	-0.28, 0.85	0.324	
Sex, male	-0.10	-0.20, 0.01	0.067	
Age, years	-0.03	-0.04, -0.02	**<0.001**	
Education, years	0.02	0.01, 0.04	**0.001**	
Gross income, 5000 €	0.01	-0.00, 0.02	0.256	
Learning and memory 2011	-0.30	-0.35, -0.25	**<0.001**	
Δ *Reaction time*				0.39
(Intercept)	1.02	0.58, 1.46	**<0.001**	
Sex, male	-0.06	-0.14, 0.02	0.148	
Age, years	-0.02	-0.03, -0.02	**<0.001**	
Education, years	0.00	-0.01, 0.01	0.586	
Gross income, 5000 €	0.01	0.00, 0.02	**0.018**	
Reaction time 2011	-0.59	-0.63, -0.55	**<0.001**	
Δ *Information processing*				0.11
(Intercept)	0.14	-0.31, 0.60	0.534	
Sex, male	0.11	0.02, 0.19	**0.012**	
Age, years	-0.02	-0.03, -0.01	**<0.001**	
Education, years	0.04	0.03, 0.05	**<0.001**	
Gross income, 5000 €	0.01	0.00, 0.02	**0.037**	
Information processing 2011	-0.24	-0.28, -0.20	**<0.001**	
Δ *Working memory*				0.039
(Intercept)	1.27	0.20, 2.33	**0.020**	
Sex, male	0.37	0.17, 0.56	**<0.001**	
Age, years	-0.06	-0.08, -0.04	**<0.001**	
Education, years	0.03	-0.00, 0.05	0.059	
Gross income, 5000 €	0.02	-0.00, 0.04	0.111	
Working memory 2011	-0.13	-0.23, -0.03	**0.008**	

Δ: Delta change of cognitive performance between years 2018 and 2011. Values are β estimates and their 95% confidence intervals and *p* values from linear models. All variables are entered simultaneously into the model. Gross yearly income was reported as 5000 € intervals (the highest group > 100 000 €). R^2 values represent the goodness of fit of each full model. Statistically significant *p* values are bolded.
